# Sharpin Contributes to TNFα Dependent NFκB Activation and Anti-Apoptotic Signalling in Hepatocytes

**DOI:** 10.1371/journal.pone.0029993

**Published:** 2012-01-09

**Authors:** Sabrina Sieber, Nicole Lange, Gwendlyn Kollmorgen, Annette Erhardt, Alexander Quaas, Arthur Gontarewicz, Gabriele Sass, Gisa Tiegs, Hans-Jürgen Kreienkamp

**Affiliations:** 1 Institut für Humangenetik, Universitätsklinikum Hamburg-Eppendorf, Hamburg, Germany; 2 Institut für Experimentelle Immunologie und Hepatologie, Universitätsklinikum Hamburg-Eppendorf, Hamburg, Germany; 3 Institut für Pathologie, Universitätsklinikum Hamburg-Eppendorf, Hamburg, Germany; University of Hong Kong, Hong Kong

## Abstract

TNFα stimulates both pro- and anti-apoptotic signalling in hepatocytes. Anti-apoptotic signalling depends on a cascade of ubiquitylation steps leading to NFκB activation. Using Sharpin-deficient mice, we show that the ubiquitin binding protein Sharpin interacts with Hoip, an E3 ligase which generates linear ubiquitin chains. Sharpin-deficiency sensitized hepatocytes to induction of apoptosis by TNFα even in the absence of transcriptional inhibition. TNFα induced activation of NFκB was strongly reduced in hepatocytes from Sharpin-deficient mice, due to reduced and delayed phosphorylation and degradation of IκBα. Injection of TNFα-inducing lipopolysaccharides led to strongly exacerbated liver damage and premature death in Sharpin-deficient mice. Our findings point to an essential role of Sharpin in linear ubiquitin chain formation, NFκB activation, and protection of the liver against inflammatory damaging signals.

## Introduction

Signalling through the NFκB pathway controls cellular events including inflammation, proliferation and apoptosis. In hepatocytes, activation of NFκB is induced by TNFα and constitutes an anti-apoptotic signal, which counterbalances the pro-apoptotic branch of TNFα dependent signalling initiated by activation of caspase-8 [1]–[3]. NFκB activation relies on a cascade of ubiquitylation events which trigger activation or degradation of signalling intermediates. The type of linkage determines the specificity for ubiquitin chain dependent signalling [4]. Besides internal lysine residues K48 and K63, which have been widely studied, linear (N- to C-terminal) ubiquitin chains are essential for activation of NFκB [5]. Formation of linear chains is catalyzed by a linear ubiquitin chain assembly complex (LUBAC) made up of E3 ligases Hoip and HOIL-1L [6]. Both proteins contain several ubiquitin binding zinc finger motifs of the Npl4 type (NZF). Interaction between HOIL-1L and Hoip is mediated by a ubiquitin like (Ubl) domain in HOIL-1L and a ubiquitin associated (UBA) motif in Hoip. During incubation of cells with TNFα, LUBAC is recruited to the TNF receptor complex [7] and ubiquitylates the NFκB essential modulator, NEMO [8].

Interestingly, HOIL-1L has a close homolog named Sharpin which shares two functional domains with HOIL-1L, the Ubl domain and the NZF-type zinc finger. Sharpin is not an E3 ligase as it lacks the *really interesting new gene* motifs (RING fingers) present in HOIL-1L and Hoip. Sharpin was initially discovered as an interaction partner of postsynaptic Shank proteins [9]. However, expression of Sharpin is not limited to neuronal tissues, indicating that Sharpin fulfills non-synaptic functions. Seymour et al. [10] described a loss-of-function mutation in the mouse *Sharpin* gene which causes a phenotype termed chronic proliferative dermatitis mutation (cpdm). Mice suffer from eosinophilic dermatitis, multiple organ inflammation, absence of Peyer's patches, splenomegaly and abnormal lymphoid architecture [11]. Initial studies in the skin of Sharpin-deficient mice pointed to alterations in NFκB signalling consistent with a constitutive activation of NFκB [12]. More recently, however, it became clear that Sharpin is a component of the LUBAC complex and contributes to TNFα-induced activation of NFκB [13]–[15]. Here we show that Sharpin is involved in canonical NFκB activation in hepatocytes pointing to a critical role of Sharpin for liver cell survival. TNFα induced activation of NFκB was found strongly reduced in hepatocytes from Sharpin-deficient mice, due to diminished and delayed phosphorylation and degradation of IκBα. This led to increased apoptosis upon TNFα treatment. Since the liver is continuously exposed to food antigens and bacterial toxins from the gut via the portal vein, inflammatory mediators such as TNFα are constantly produced in this organ. Being one component of the LUBAC complex, Sharpin seems to be part of the hepatocellular defence, favouring anti-apoptotic signalling and thereby counteracting liver damage under physiological conditions.

## Results

**Figure 1:**
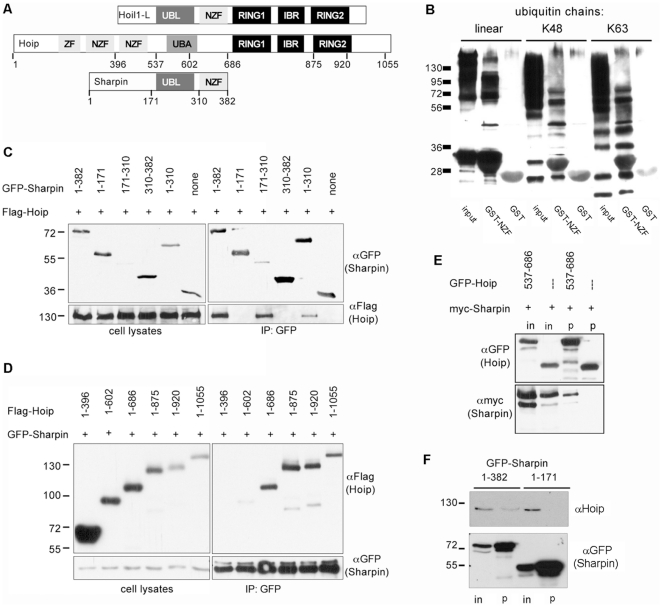
Sharpin interacts with ubiquitin and the E3 ubiquitin ligase Hoip. A. Domain structure of HOIL-1L, Hoip and Sharpin. Sharpin is similar to HOIL-1L in its Ubl and NZF domains but lacks the Ring/IBR/Ring E3 ligase motif. For Hoip and Sharpin, positions used to generate deletion constructs are indicated. B. The NZF domain of Sharpin binds ubiquitin. An immobilized GST fusion protein of the NZF domain (or GST alone) was incubated with linear, K48 or K63 linked polyubiquitin chains; after centrifugation and washing, input and precipitate samples were analyzed by Western blotting with anti-ubiquitin. C. The Ubl domain of Sharpin binds to Hoip. GFP-fusion proteins of Sharpin or fragments thereof were coexpressed with Flag-tagged Hoip in HEK293 cells. Cell lysates were subjected to immunoprecipitation of GFP containing proteins using GFP-Trap matrix, and input (left) and precipitate (right) samples were analyzed by Western blotting using anti-GFP (upper panels) and anti-Flag (lower panels) antibodies. Note that the isolated Ubl domain (residues 171–310) of Sharpin precipitates Hoip very efficiently despite low expression levels. D. The central region of Hoip mediates interaction with Sharpin. Flag-tagged fragments of Hoip were coexpressed with GFP-Sharpin, and subjected to immunoprecipitation of GFP, as in C. E. The UBA domain of Hoip is sufficient for interaction with Sharpin. Myc-tagged Sharpin was coexpressed with a GFP-Hoip encompassing the UBA domain, or GFP alone. Cell lysates were subjected to immunoprecipitation of GFP and input (in) and precipitate (p) samples were analyzed by Western blotting. F. Interaction in hepatocarcinoma cells. Constructs encoding GFP as a fusion with full length Sharpin (amino acids 1–382), or only the N-terminal region (residues 1–171) were expressed in Huh-7 hepatocarcinoma cells. After cell lysis, GFP-containing proteins were immunoprecipitated using GFP-Trap matrix. Input (in) and precipitate (p) samples were analyzed by Western blotting using antibodies against human Hoip (upper panel) or GFP (lower panel).

The domain structures of Hoip, HOIL-1L and Sharpin are shown in Figure 1A. The similarity of Sharpin to HOIL-1L indicates that Sharpin might also bind to ubiquitin chains via the NZF domain and to Hoip, an ubiquitin E3 ligase of the LUBAC complex. An interaction with ubiquitin was demonstrated by preparing a GST fusion protein of the NZF domain and incubation with different types of ubiquitin chains. Here we observed that the Sharpin NZF domain specifically associated with K48 and K63 linked chains as well as linear chains (Fig. 1B).

To test for an interaction with Hoip, GFP-tagged Sharpin and Flag-tagged Hoip were expressed in HEK293 cells. When Sharpin was immunoprecipitated from these cells via the GFP-tag, Hoip could be efficiently coprecipitated (Fig. 1C). By using deletion constructs we could identify the Ubl domain of Sharpin as the motif responsible for this interaction. On the other hand, a series of deletion constructs for Hoip was tested for interaction with full-length Sharpin (Fig. 1D). Here a fragment containing residues 1–686 showed efficient interaction, whereas residues 1–396 did not interact. Interestingly, residues 1–602 showed a rather weak interaction. As this fragment contains most but not all amino acids of the UBA domain, we assumed that the UBA domain mediates the interaction on behalf of Hoip. This was confirmed by coexpressing myc-tagged Sharpin with a GFP fusion protein of the UBA domain of Hoip only (residues 537–686). Precipitation of this GFP fusion, but not of GFP alone, led to efficient coprecipitation of Sharpin (Fig. 1E). Furthermore, overexpression and immunoprecipitation of GFP-Sharpin leads to efficient coprecipitation of endogenous Hoip expressed in the human Huh-7 hepatocarcinoma cell line (Fig. 1F).

**Figure 2:**
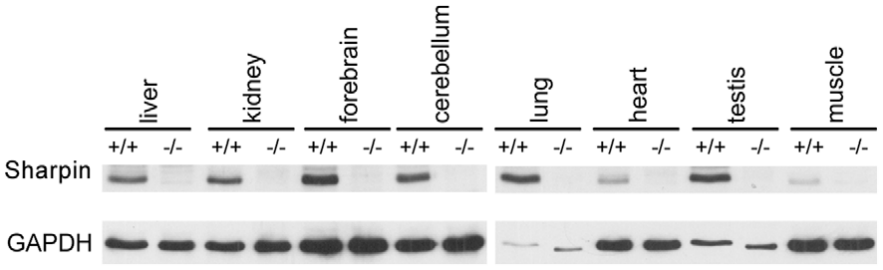
Sharpin is ubiquitously expressed in mouse tissues. The tissues indicated were prepared from wt and Sharpin-deficient mice; after lysis in RIPA buffer and centrifugation, clear supernatants were analyzed by Western blotting using anti-Sharpin (upper panels) or anti-GAPDH (lower panels). WT mice (^+/+^); Sharpin-deficient mice (^−/−^).

**Figure 3:**
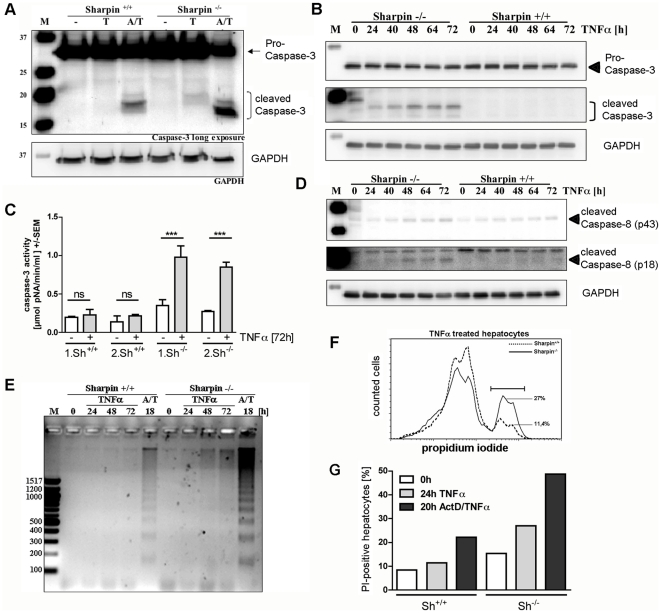
TNFα induces apoptosis in hepatocytes from Sharpin-deficient mice. A. Activation of caspase-3. Hepatocytes were isolated from wt and Sharpin-deficient mice. After 24 hours in culture, cells were incubated with TNFα alone for 24 hours (T) or in combination with ActD (A/T) for 18 hours. Cell lysates were analyzed by Western blotting with an antibody against caspase-3 (top) or GAPDH (bottom). Caspase-3 blots were subjected to long exposure to monitor appearance of the small, active fragments of caspase-3. B. Time course of caspase-3 cleavage. Hepatocytes isolated as described in A were incubated with TNFα for the times indicated. Cell lysates were analyzed by Western blotting with caspase-3 and GAPDH antibodies. C. Caspase-3 enzyme activity. Hepatocytes from two wt and two Sharpin-deficient mice were incubated with TNFα, and caspase-3 activity was monitored in quadruplicate for each animal using a caspase-3 assay kit. ANOVA/Tukey test; ***, p<0.0001, ns.-not significant. D. Time course of caspase-8 cleavage. Hepatocytes from wt and Sharpin-deficient mice were incubated as described in B and cell lysates were analyzed by Western blotting with an antibody recognizing the cleaved intermediate (p43) and active (p18) forms of caspase-8. E. DNA-fragmentation. Hepatocytes were incubated with TNFα for the times indicated or in combination with ActD (A/T) for 18 hours; genomic DNA was analyzed, and DNA-fragmentation was analyzed by agarose gel electrophoresis. F, G. Quantification of PI-positive cells. FACS analysis of hepatocytes from wt and Sharpin-deficient cells after incubation with TNFα for 24 hours or ActD/TNFα for 20 hours. In F, Histogram of propidium iodide (PI) intensity indicating PI-positive wt (dotted line; 11.4%) and Sharpin-deficient (solid line; 27%) hepatocytes after 24 hours of TNFα incubation. G. Relative proportion of PI-positive cells after incubation with TNFα or ActD/TNFα. Equal numbers of cells are depicted for all experiments. One typical of two experiments is shown. WT mice (^+/+^); Sharpin-deficient mice (^−/−^).

Both HOIL-1L and Hoip are widely expressed. Similarly, as shown here by Western blotting, Sharpin was found to be expressed in all tissues analyzed, with low levels in heart and skeletal muscle tissue. Tissues from Sharpin-deficient mice were used as negative control for antibody staining (Fig. 2). Previous experiments have shown that deficiency of HOIL-1L interferes with NFκB activation and leads to enhanced apoptosis in hepatocytes after TNFα incubation [8], and that Sharpin contributes to TNFα signalling in various cell types [13]–[15]. Therefore we asked whether the absence of Sharpin also contributes to TNFα induced apoptosis in the liver. Apoptosis of hepatocytes can be induced by TNFα in combination with Actinomycin D (ActD). Thus, the anti-apoptotic branch of TNFα signalling is blocked by inhibiting transcription, whereas the pro-apoptotic caspase-8/caspase-3 cascade remains unaffected. Hepatocytes from wild type (wt) and Sharpin-deficient mice were incubated with TNFα alone, or with ActD/TNFα. Analysis of caspase-3 activation indicated that apoptosis was induced in hepatocytes from Sharpin-deficient mice by the ActD/TNFα combination and, to a weaker extent, in hepatocytes from wt mice. Strikingly, treatment with TNFα alone induced cleavage of caspase-3 in cells from Sharpin-deficient, but not wt mice (Fig. 3A). Even after prolonged times of incubation, little caspase-3 activation was observed in wt hepatocytes incubated with TNFα alone, while the appearance of the active form of caspase-3 was readily observed in Sharpin-deficient cells after 24–72 hours (Fig. 3B). Quantification of caspase-3 enzyme activity confirmed these data (Fig. 3C). By monitoring the activity of caspase-8 (which is upstream of caspase-3) we observed that the smaller, active fragments of caspase-8 appeared in hepatocytes from Sharpin-deficient, but not wt cells (Fig. 3D). Furthermore, we showed that incubation with TNFα alone induced fragmentation of genomic DNA in Sharpin-deficient, but not wt hepatocytes. Cells from both genotypes showed DNA-fragmentation when incubated with the ActD/TNFα combination, as expected (Fig. 3E). In addition, we performed FACS analysis of TNFα treated hepatocytes with the membrane impermeable dye propidium iodide (PI; Fig. 3F,G). Here we observed that the number of PI-positive hepatocytes increased only marginally in TNFα incubated wt cells (8.3% in non-incubated to 11.4% in incubated cells), whereas the frequency of PI-positive Sharpin-deficient hepatocytes nearly doubled upon TNFα incubation (15.3% to 27%). As expected, incubation of wt and Sharpin-deficient hepatocytes with ActD/TNFα as a control showed an increase of PI-positive cells in both samples (Fig. 3G).

**Figure 4:**
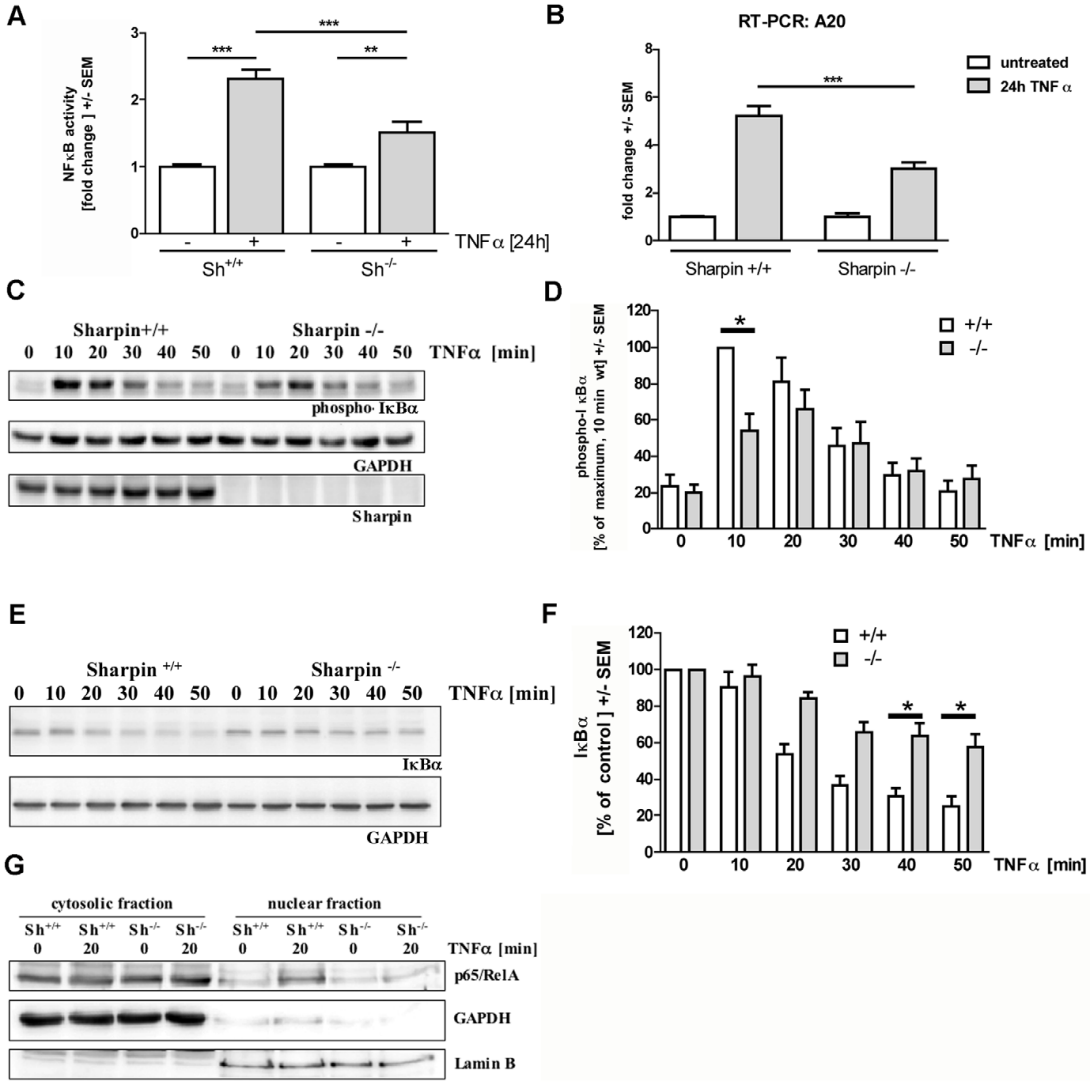
Reduced NFκB activation by TNFα in primary hepatocytes from Sharpin-deficient mice. A. NFκB activity assay. Hepatocytes isolated from wt and Sharpin-deficient mice were transfected with reporter vectors encoding CMV-driven *Renilla* luciferase and NFκB promoter dependent *Photinus* luciferase. After incubation with TNFα for 24 hours, cell lysates were analyzed for activity of both luciferases; NFκB promoter activity is depicted as the ratio between *Photinus* and *Renilla* luciferase activities from three independent experiments. ANOVA/Tukey test **,*** p<0.01/0.001, respectively; n = 3. B. Real time RT-PCR analysis. Hepatocytes isolated from wt and Sharpin-deficient mice were incubated with TNFα for 24 hours. After isolation of RNA, levels of the mRNA coding for A20 were determined by quantitative real time RT-PCR relative to levels of mRNA coding for ATP synthase beta. C,D. Phosphorylation of IκBα. Hepatocytes were incubated with TNFα for the times indicated. Cell lysates were analyzed by Western blotting using the antibodies indicated (C). In D, the amount of phospho-IκBα from three independent experiments as shown in C is quantified. The intensity of phospho-IκBα signals was normalized on GAPDH signals; in a second step, data were normalized on the maximum value (in each case the 10 minute time point in wt cells). *, +/+ data significantly different from −/− data; p<0.05; n = 3; t-test. E,F. Degradation of IκBα. Hepatocytes were incubated as described in C, and analyzed by Western blotting with the antibodies indicated. In F, the amount of the amount of IκBα from three independent experiments as shown in E is quantified. In this case the amount of IκBα in the control experiment (no TNFα incubation) was set as 100% for each genotype. G. Nuclear translocation of NFκB. Hepatocytes were incubated with TNFα as indicated; cytosolic and nuclear fractions were prepared and analyzed by Western blotting with antibodies against GAPDH (cytosolic marker), Lamin B (nuclear marker) and p65/RelA. Note that the intensity of the signal for the p65/RelA component of NFκB increases in nuclei of TNFα-incubated wt, but not Sharpin-deficient cells. WT mice (^+/+^); Sharpin-deficient mice (^−/−^).

Our data indicated that caspase-dependent pro-apoptotic signalling is activated by ActD/TNFα in wt and Sharpin-deficient hepatocytes, whereas the anti-apoptotic, transcription dependent pathway is partially lost in Sharpin-deficient cells. Therefore we analyzed whether NFκB activation is altered in cells lacking Sharpin. A reporter vector encoding the firefly (*Photinus*) luciferase coding sequence under control of NFκB promoter elements was cotransfected into primary hepatocytes in combination with a CMV promoter driven *Renilla* luciferase plasmid. Cells were again incubated with TNFα and analyzed for both luciferase activities. Here we observed that activation of the NFκB dependent promoter was significantly stronger in wt cells (2.3-fold) than in Sharpin-deficient hepatocytes (1.5-fold; Fig. 4A). This was mirrored in the transcriptional response of the NFκB responsive gene coding for A20, a negative regulator of NFκB signalling. Analysis by quantitative real time RT-PCR showed that TNFα incubation increased the cellular levels of A20 mRNA more than 5.3-fold in wt cells, but only 3-fold in Sharpin-deficient cells (Fig. 4B). For a more detailed analysis we focused on the phosphorylation and subsequent degradation of IκBα. In wt cells, we observed rapid phosphorylation of IκBα within 10 minutes of incubation with TNFα (Fig. 4C,D). Levels of phospho-IκBα then declined concomitantly with a decrease of overall IκBα levels (Fig. 4E,F). In Sharpin-deficient cells, TNFα induced phosphorylation was detectable but the signal intensity at the 10 minute time point was significantly decreased. Maximum phosphorylation levels were observed not until 20 minutes of incubation with TNFα, where they matched those obtained in wt cells. Phosphorylation is the key signal leading to degradation of IκBα; in agreement, we observed that degradation of IκBα was delayed and incomplete in Sharpin-deficient cells (Fig. 4E,F). Importantly, delayed degradation was associated with a failure of the active transcription factor p65/RelA to translocate to the nucleus in Sharpin-deficient cells upon TNFα incubation (Figure 4G).

**Figure 5:**
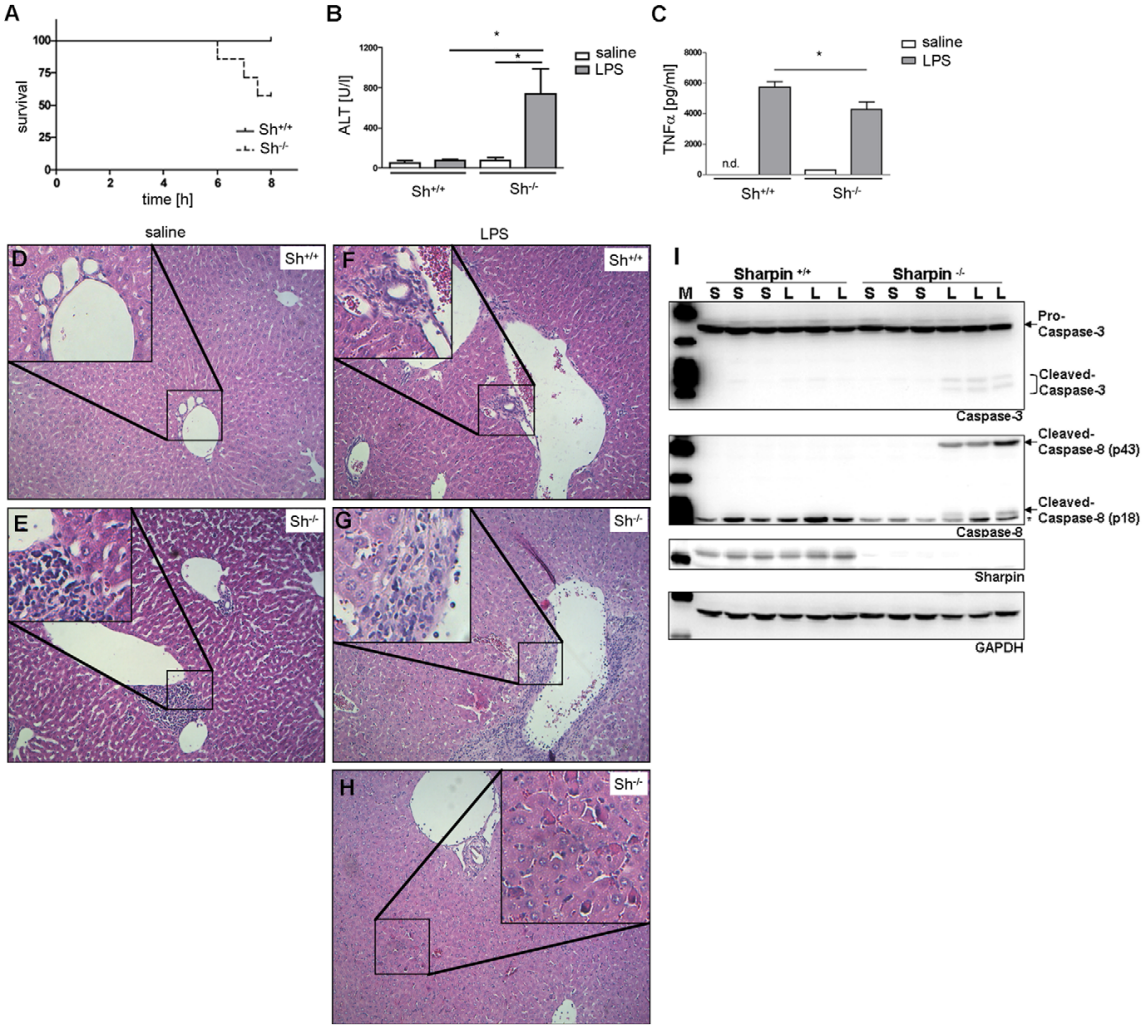
LPS induces severe liver failure in Sharpin-deficient mice. Wt and Sharpin-deficient mice (age: 9 weeks) were injected with LPS (4 mg/kg i.p.; 7 animals per genotype; 4 animals per genotype in saline injected control group). A. Survival curve. Three out of seven Sharpin-deficient mice died within the first 8 hours after injection. B. Serum ALT activity. Surviving animals were sacrificed after 8 hours and analyzed for serum alanine aminotransferase (ALT) levels. C. TNFα release. Serum TNFα levels were determined 1 hour after LPS treatment from all mice (including those which died within the first 8 hours) by ELISA. D–H. Pathological examination after 8 hours of treatment. Liver sections were H&E stained. Note the presence of lymphocytic infiltrates in liver from Sharpin-deficient mice (E,G) as well as necrosis after LPS injection (G,H). I. Activation of the caspase cascade. Liver tissue lysates of saline (S) and LPS (L) treated mice were analyzed by Western blotting using Caspase-3 and Caspase-8 specific antibodies, as described in Figure 3. Note the appearance of cleaved caspases in LPS treated Sharpin-deficient mice only (unspecific band marked with an asterisk).

Finally, we investigated the relevance of Sharpin for TNFα mediated inflammatory responses in the liver *in vivo*. Injection of lipopolysaccharide (LPS) is known to induce TNFα release from Kupffer cells, which causes hepatocyte cell death in presence of transcriptional inhibition [16]. Importantly, injection of a single dose of LPS alone led to acute liver damage (as determined by serum alanine aminotransferase levels [ALT]) and in several cases death in Sharpin-deficient mice, whereas control mice exhibited no increase in serum ALT activity (Fig. 5A,B). As expected, both wt and Sharpin-deficient mice exhibited a strong increase in serum TNFα levels, which was, however, less pronounced in Sharpin-deficient mice (Fig. 5C). This may be due to the fact that the gene coding for TNFα is itself a target gene of NFκB. As observed before [17], our histological analysis showed severe inflammatory reaction with predominant lymphocytic infiltration in sections obtained from Sharpin-deficient mice, whereas normal liver structure and no inflammatory infiltrates were found in wt mice. After LPS injection, liver tissue from wt mice exhibited small inflammatory infiltrates. In Sharpin-deficient animals, we detected again a severe inflammatory reaction with lymphocyte infiltration. In addition we observed necrotic and apoptotic cell death of hepatocytes (Fig. 5D–H). Furthermore, analysis of liver tissue lysates showed the activation of the caspase-8/caspase-3 cascade. We detected the cleaved and active forms of both caspase-8 and caspase-3 in LPS treated Sharpin-deficient mice, whereas LPS treated wt mice remained unaffected (Fig. 5I). These results are in agreement with the massive increase in serum ALT levels in Sharpin-deficient mice.

## Discussion

Our data extend previous evidence showing that Sharpin, by virtue of its interaction with the ubiquitin E3 ligase Hoip, is a component of the linear ubiquitin chain assembly complex LUBAC [13]–[15]. Sharpin is highly similar to HOIL-1L, another E3 ligase, which together with Hoip is responsible for the attachment of linear ubiquitin chains to target proteins [5], [6]. The similarity between HOIL-1L and Sharpin is limited to the Ubl and NZF domains. In both proteins the Ubl domains mediate interaction with Hoip; in contrast to results by Ikeda et al. [14] who suggested that Hoip uses its NZF domain to interact with Sharpin, we find here that the UBA domain of Hoip is responsible for the interaction, in agreement with [15]. The UBA domain of Hoip is also required for interaction with HOIL-1L [8], pointing to a common mode of interaction. Apparently the NZF domains of Sharpin and HOIL-1L fulfill similar functions as both bind to ubiquitin chains (Fig. 1B; see also [13]–[15]). In addition, Sharpin contains an N-terminal region of unknown function.

Recent work has shown that the E3 activity of Hoip (but not of HOIL-1L) is responsible for activity of the complex. A truncated HOIL-1L protein lacking the E3 ligase domain could functionally replace full-length HOIL-1L [6], [8]. Sharpin does not carry an E3 ligase domain, and it is likely that Sharpin is a structural, but not a catalytic component of the LUBAC complex. We show here that in hepatocytes Sharpin is required for efficient signal transduction from TNFα to NFκB. In Sharpin-deficient hepatocytes, incubation with TNFα alone results in increased apoptosis, as shown by DNA-fragmentation and PI-staining. Recently, similar results were obtained in primary keratinocytes (13). We further show that increased apoptosis is due to activation of the caspase-8/caspase-3 cascade. In wt hepatocytes TNFα induces a survival signal via NFκB-dependent expression of anti-apoptotic proteins which is inhibited by incubation with the transcriptional inhibitor ActD [18], [19]. We observed an even increased level of TNFα-induced caspase-3 activation in Sharpin-deficient vs. wt hepatocytes in presence of ActD, suggesting that ActD only incompletely inhibited hepatocellular transcription still giving rise to Sharpin-dependent survival signals.

The increase in apoptosis in hepatocytes lacking Sharpin is due to a failure in fast and complete activation of the canonical NFκB pathway upon TNFα incubation. Phosphorylation and degradation of IκBα are reduced and delayed in Sharpin-deficient hepatocytes, and consequently NFκB is unable to translocate to the nucleus and activate transcription after TNFα incubation. Thus the anti-apoptotic branch of the TNFα signal is attenuated, tipping the balance towards apoptosis.

Our data are in contrast to work by Liang et al. [12], who observed constitutive activity of NFκB in the skin of Sharpin-deficient mice. At present it is not easy to reconcile these differences. However, Gijbels et al. analyzed several organs of Sharpin-deficient mice and found that cellular infiltration of eosinophils is not limited to the skin but also affects several other organs [17]. Therefore, NFκB activation in the skin may be secondary to the strong inflammatory reaction in Sharpin-deficient mice.

It should be noted that NFκB activation is not lost completely. Both Sharpin and HOIL-1L contribute to full LUBAC activity and may be important for substrate recognition or subcellular targeting of the complex. Similar to Sharpin-deficient mice, HOIL-1L knockout mice are viable but exhibit TNFα-induced apoptosis in hepatocytes [8]. Walczak and coworkers [7], [13] reported that LUBAC is recruited to the TNF-receptor upon activation; the delay in IκBα phosphorylation in Sharpin-deficient cells might be due to insufficient recruitment of LUBAC to the TNF-receptor complex and point to a role for Sharpin in the targeting of LUBAC to the TNF-receptor.

NFκB signalling is one of the most important survival pathways of hepatocytes; mice lacking the p65/RelA component of NFκB die *in utero* due to hepatocyte apoptosis and liver degeneration [20]. NFκB activation is also required to protect the adult liver from acute and chronic injury, since hepatocyte specific NEMO knockout mice showed increased sensitivity to TNFα-induced liver damage, developed a phenotype of spontaneous chronic hepatitis associated with increased hepatocyte apoptosis, liver inflammation, steatosis, compensatory hepatocyte proliferation and finally development of hepatocellular carcinoma [21]. The relevance of this pathway for protection of hepatocytes against inflammation is illustrated by our *in vivo* experiments, where we observe massive liver damage, necrosis and death in Sharpin-deficient mice upon LPS injection. Therefore it seems that this pathway is safeguarded in hepatocytes by different, more or less redundant, activation steps in the canonical activation pathway leading to IκBα degradation and translocation of NFκB. Finally, compared to the widely used liver injury models depending on transcriptional inhibition, Sharpin-deficient mice might represent a model to study mechanisms and consequences of failure in this pathway being more related to injury-dependent liver pathology.

## Materials and Methods

### Ethics Statement

All animal experiments were performed according to local regulations of the state authorities in Hamburg, Germany. In particular, permission was obtained from “Amt für Gesundheits- und Verbraucherschutz” of “Behörde für Soziales, Familie, Gesundheit und Verbraucherschutz”, State Government of Freie und Hansestadt Hamburg, for perfusion of the liver and removal of hepatocytes from anaesthetized mice (registration number G 21301/591-00.33, obtained on September 1, 2009). Permission for injections of LPS into wt and Sharpin-deficient mice was obtained with the registration number 105/10, Behörde für Soziales, Familie, Gesundheit und Verbraucherschutz, Hamburg, 20.01.2011 and 31.03.2011.

### Mice

Sharpin-deficient (cpdm) mice were obtained from The Jackson Laboratories and maintained as heterozygous mice on C57/Bl6 background. For genotyping, the genomic region encompassing the one base pair deletion in exon 1 of the *Sharpin* gene which leads to the cpdm phenotype was amplified from genomic DNA by PCR using primers CGGACCGTGCCTGGAG and CACGAATGTGAAAGGAAAAG, and sequenced using GATCAGAAATGTCGCCGC.

### Antibodies and reagents

Anti-Sharpin was generated by custom immunization of rabbits using a GST-fusion protein of the N-terminal domain (residues 1–171) of rat Sharpin by Biogenes GmbH (Berlin, Germany). Antibodies against IκBα, P-IκBα, Caspase-3 (8G10), Cleaved Caspase-8 (Asp387) and GAPDH were obtained from Cell Signaling Technologies (New England Biolabs, Frankfurt am Main, Germany); anti-GFP from Chromotek (Munich, Germany); anti-Flag from Sigma-Aldrich (Munich, Germany); anti-ubiquitin from Chemicon (Millipore, Schwalbach, Germany); anti-Hoip from Abcam (Cambridge, USA) and anti-laminB from Progen Biotechnik (Heidelberg, Germany). Recombinant mouse TNFα was from SINO Biological Inc. (Beijing, China), Actinomycin D (ActD) from Th. Geyer (Hamburg, Germany). Caspase-3 activity was measured using a Colorimetric Assay Kit (Sigma-Aldrich); luciferase activity was determined using the Dual-Luciferase® Reporter Assay System (Promega, Mannheim, Germany).

### GST pulldown assay

For expressing a GST fusion of the NZF domain of rat Sharpin, a cDNA fragment coding for residues 310–382 was cloned into pGEX4T2; protein was expressed in *E.coli* strain BL21, purified using glutathione sepharose (GE Healthcare, Munich, Germany) and left on the beads. For pulldown assays with ubiquitin chains (Boston Biochem, Boston, MA), GST-NZF or GST alone was incubated with ubiquitin chains in 150 mM NaCl; 10 µM ZnCl_2_; 20 mM Tris, pH 6.8; 5 mM β-mercaptoethanol; 0.1% Triton-X 100 for 2 hours at 4°C. After washing, samples were analyzed by Western blotting.

### Expression in human cell lines, immunoprecipitation

A cDNA coding for rat Sharpin was obtained from Dr. Eunjoon Kim (Daejon, South Korea). cDNA fragments were amplified by PCR with primers carrying appropriate restriction sites and cloned into pEGFP-C vectors in frame with the EGFP coding sequence. A cDNA coding for Hoip was obtained from Origene (Rockville, USA); the coding region was cloned into pCMV2B, leading to expression of N-terminally Flag-tagged Hoip. For the generation of deletion constructs we took advantage of existing restriction sites in the Hoip cDNA sequence. For expression, HEK293 cells were cotransfected with pEGFP-Sharpin and pCMV-Hoip plasmids using Turbofect (Fermentas GmbH, St. Leon-Rot, Germany). Cells were lysed in RIPA buffer and lysates were cleared by centrifugation (20400× g; 4°C; 20 min). GFP-containing fusion proteins were precipitated with GFP-trap matrix (Chromotek). After washing, samples were analyzed by Western blotting. Huh-7 cells were transfected with GFP-Sharpin using Turbofect, followed by lysis and immunoprecipitation as described for HEK293 cells.

### Preparation of primary hepatocytes

Hepatocytes were isolated from 6 to 9 weeks old mice by a modification of the 2-step collagenase perfusion method of Seglen [22], [23] followed by 10 min centrifugation in a 90% Percoll gradient (GE Healthcare) at 50× g to obtain highly purified hepatocytes. For culture of hepatocytes, William's E+GlutaMAX™-I medium (Gibco, Karlsruhe, Germany) was supplemented with 10% FBS (Gibco), 1% penicillin/streptomycin (Biochrom AG seromed, Berlin, Germany) and 1% L-glutamine (Invitrogen-GIBCO, Karlsruhe, Germany). Hepatocytes were transfected using Lipofectamin 2000 (Invitrogen, Darmstadt, Germany) according to the manufacturer's protocol. For activation of NFκB and/or induction of apoptosis hepatocytes were incubated either with 40 ng/ml TNFα alone or with ActD (100 nM) and TNFα (40 ng/ml) as described previously [22]. For Western Blot analysis, cells were lysed in 50 mM Tris/HCl (pH 8.0); 150 mM NaCl; 1% NP40; 0.5% Na-Deoxycholat; 5 mM EDTA; 0.1% SDS; 1× Phosphatase Inhibitor Cocktail (Roche Applied Sience, Mannheim, Germany) and 1× Protease Inhibitor Cocktail (Sigma-Aldrich). For Western Blot analysis of mouse liver tissue, protein lysates were prepared as described previously [19]. For DNA-fragmentation analysis, cells were lysed in 10 mM Tris/HCL pH 8; 1 mM EDTA and 0.2% Triton X-100. After centrifugation the low molecular weight DNA-containing supernatant was digested with RNase A for 1 hour at 37°C (final concentration 60 µg/ml). Subsequently, SDS (final concentration 0.5%) and Proteinase K (final concentration 150 µg/ml) were added and incubated for 1 hour at 50°C. After that DNA was precipitated with isopropanol, dissolved in 10 mM Tris/HCL pH 8; 1 mM EDTA and analyzed on a 2% agarose gel.

For separation of cytosolic and nuclear fractions, cells were lysed in 10 mM HEPES pH 7.8; 10 mM KCl; 0.1 mM EDTA and 1× Protease Inhibitor Cocktail (Sigma-Aldrich) for 20 min. NP-40 (final concentration 0.5%) was added and cells were incubated for 5 min. After centrifugation (5000× g) the supernatant, containing the cytosolic fraction, was harvested. The nuclei-containing pellet was resuspended in 20 mM HEPES pH 7.8; 500 mM KCl; 1 mM EDTA; 10% Glycerol; 1 mM DTT and 1× Protease Inhibitor Cocktail (Sigma-Aldrich) and lysed for 30 min. After centrifugation (12000× g) the supernatant, containing the nuclear proteins, was harvested. Both fractions were analyzed by Western blotting.

### Flow cytometric analysis

Primary hepatocytes derived from 7 weeks old male mice were cultured and stimulated as described above and harvested by trypsinisation. A total of 5×10^5^ hepatocytes were stained using standard Annexin V (Alexa Fluor® 647) and Propidium Iodid (PI) staining protocol (Biolegend, Fell, Germany). Data were recorded using the BD FACSCanto II system and BD FACSDiva software (BD Biosciences, Heidelberg, Germany) and analyzed with FCS express 4 (DeNovo Software, Los Angeles, USA).

### Detection of mRNA by Real Time RT-PCR

Total RNA was extracted from hepatocytes using TRIzol reagent (Invitrogen) and reverse transcribed using the Verso cDNA synthesis Kit (Abgene, Epsom, UK) according to the manufacturer's protocol. Oligonucleotides for PCR reactions were obtained from Metabion International AG (Martinsried, Germany) with the following sequences (5′→3′): A20 forward: CCAGGTTCCAGAACAATGTC, A20 reverse: CTCCATACAGAGTTCCTCAC, ATPsynthase beta forward: ATTGCCATCTTGGGTATGGA, ATPsynthase beta reverse: AATGGGTCCCACCATGTAGA. Real time RT-PCR was performed using the C1000 Thermal Cycler+CFX 96 Real-Time System (Bio-Rad, Munich, Germany) and the MaximaTM SYBR Green qPCR Master Mix (Fermentas GmbH). Reactions were carried out in a 10 µl volume.

### Application of LPS *in vivo*

Lipopolysaccharide (LPS) from *Salmonella abortus equi* was purchased from Sigma-Aldrich and administered i.p. at a concentration of 4 mg/kg. Hepatocyte damage was assessed 8 hours after LPS administration by measuring plasma enzyme activities of alanine aminotransferase (ALT) using an automated procedure (COBAS MIRA®; Roche, Switzerland; [24]). Plasma TNFα levels were measured by sandwich ELISA as described previously [25].

### Histology

Liver tissue from mice was fixed in 4% phosphate-buffered formalin and embedded into paraffin. 3 µm paraffin sections were H&E stained and basic histomorphology of the specimens was evaluated.

## References

[1] Karin M, Greten FR (2005). NF-κB: linking inflammation and immunity to cancer development and progression.. Nat Rev Immunol.

[2] Hayden MS, Ghosh S (2008). Shared principles in NF-κB signaling.. Cell.

[3] Pasparakis M (2009). Regulation of tissue homeostasis by NF-κB signalling: implications for inflammatory diseases.. Nat Rev Immunol.

[4] Ikeda F, Crosetto N, Dikic I (2010). What determines the specificity and outcomes of ubiquitin signaling?. Cell.

[5] Iwai K, Tokunaga F (2009). Linear polyubiquitination: a new regulator of NF-κB activation.. EMBO Rep.

[6] Kirisako T, Kamei K, Murata S, Kato M, Fukumoto H (2006). A ubiquitin ligase complex assembles linear polyubiquitin chains.. Embo J.

[7] Haas TL, Emmerich CH, Gerlach B, Schmukle AC, Cordier SM (2009). Recruitment of the linear ubiquitin chain assembly complex stabilizes the TNF-R1 signaling complex and is required for TNF-mediated gene induction.. Mol Cell.

[8] Tokunaga F, Sakata S, Saeki Y, Satomi Y, Kirisako T (2009). Involvement of linear polyubiquitylation of NEMO in NF-κB activation.. Nat Cell Biol.

[9] Lim S, Sala C, Yoon J, Park S, Kuroda S (2001). Sharpin, a novel postsynaptic density protein that directly interacts with the shank family of proteins.. Mol Cell Neurosci.

[10] Seymour RE, Hasham MG, Cox GA, Shultz LD, Hogenesch H (2007). Spontaneous mutations in the mouse Sharpin gene result in multiorgan inflammation, immune system dysregulation and dermatitis.. Genes Immun.

[11] HogenEsch H, Janke S, Boggess D, Sundberg JP (1999). Absence of Peyer's patches and abnormal lymphoid architecture in chronic proliferative dermatitis (cpdm/cpdm) mice.. J Immunol.

[12] Liang Y, Seymour RE, Sundberg JP (2011). Inhibition of NF-κB signaling retards eosinophilic dermatitis in SHARPIN-deficient mice.. J Invest Dermatol.

[13] Gerlach B, Cordier SM, Schmukle AC, Emmerich CH, Rieser E (2011). Linear ubiquitination prevents inflammation and regulates immune signalling.. Nature.

[14] Ikeda F, Deribe YL, Skanland SS, Stieglitz B, Grabbe C (2011). SHARPIN forms a linear ubiquitin ligase complex regulating NF-κB activity and apoptosis.. Nature.

[15] Tokunaga F, Nakagawa T, Nakahara M, Saeki Y, Taniguchi M (2011). SHARPIN is a component of the NF-κB-activating linear ubiquitin chain assembly complex.. Nature.

[16] Leist M, Gantner F, Bohlinger I, Tiegs G, Germann PG (1995). Tumor necrosis factor-induced hepatocyte apoptosis precedes liver failure in experimental murine shock models.. Am J Pathol 1995.

[17] Gijbels MJ, Zurcher C, Kraal G, Elliott GR, HogenEsch H (1996). Pathogenesis of skin lesions in mice with chronic proliferative dermatitis (cpdm/cpdm).. Am J Pathol.

[18] Leist M, Gantner F, Bohlinger I, Germann PG, Tiegs G (1994). Murine hepatocyte apoptosis induced in vitro and in vivo by TNF-α requires transcriptional arrest.. J Immunol.

[19] Sass G, Shembade ND, Haimerl F, Lamoureux N, Hashemolhosseini S (2007). TNF pretreatment interferes with mitochondrial apoptosis in the mouse liver by A20-mediated down-regulation of Bax.. J Immunol.

[20] Beg AA, Sha WC, Bronson RT, Ghosh S, Baltimore D (1995). Embryonic lethality and liver degeneration in mice lacking the RelA component of NF-κB.. Nature.

[21] Luedde T, Beraza N, Kotsikoris V, van Loo G, Nenci A (2007). Deletion of NEMO/IKKγ in liver parenchymal cells causes steatohepatitis and hepatocellular carcinoma.. Cancer Cell.

[22] Haimerl F, Erhardt A, Sass G, Tiegs G (2009). Down-regulation of the de-ubiquitinating enzyme ubiquitin-specific protease 2 contributes to tumor necrosis factor-α-induced hepatocyte survival.. J Biol Chem.

[23] Seglen PO (1973). Preparation of rat liver cells. 3. Enzymatic requirements for tissue dispersion.. Exp Cell Res.

[24] Bergmeyer HU (1984). Methods *of* enzymatic analysis, 3^rd^ edition, Vol. 82.

[25] Erhardt A, Biburger M, Papadopoulos T, Tiegs G (2007). IL-10, regulatory T cells, and Kupffer cells mediate tolerance in concanavalin A-induced liver injury in mice.. Hepatology.

